# Fast Antenna Array Calibration Using One External Receiver

**DOI:** 10.3390/s23229026

**Published:** 2023-11-07

**Authors:** Basem Bazuhair, Omar Aldayel

**Affiliations:** Department of Electrical Engineering, King Saud University, Riyadh 11421, Saudi Arabia; omaldayel@ksu.edu.sa

**Keywords:** array signal processing, mutual coupling, MISO systems, massive MISO, calibration

## Abstract

In multiple array communication or radar systems, waveform diversity can be utilized for beampattern design. However, one of the critical issues for such systems is the presence of mutual coupling, which degrades the beampattern design’s quality. We address the calibration of the mutual coupling of transmit arrays by developing a new matrix-inversion-free algorithm that requires only a single antenna receiver. It has a very low computational complexity for accelerating the mutual coupling calibration compared to previous methods; therefore, it can be utilized for large array systems such as massive multiple-input–single-output (MISO) systems. The key idea here revolves around utilizing fast Fourier transform (FFT). This approach simplifies matrix calculations and reduces the number of multiplications required to compute the inverse of FFT. Moreover, the algorithm is applicable for high-power active radar calibration, since it incorporates a constant modulus training sequence. The application of the algorithm in MISO systems, including massive MISO, offers the potential for calibrating mutual coupling. It enables the precise measurement and compensation of mutual coupling effects, improving the signal quality and system performance in areas such as radars, mobile communications and more. We evaluated the proposed algorithm under various scenarios, compared it with the ground truth and showed that it achieves excellent performance with few computations.

## 1. Introduction

Smart antennas focus on electronically steering the main lobe and nulls of a radiation pattern [1]. In contrast, adaptive arrays use smart antenna concepts along with adaptive signal processing to optimize signal reception or transmission dynamically, and MIMO antennas leverage multiple antennas to achieve spatial diversity and multiplexing for enhanced data rates and link reliability [2,3]. Smart antennas, adaptive arrays and MIMO systems are crucial in modern wireless communication. They address challenges in signal quality, coverage, interference, capacity, reliability and resource allocation, meeting the increasing demand for wireless services and applications.

Phased antenna array or multiple-input–multiple-output (MIMO) systems have emerged as viable solutions to meet the rapidly growing demand for their various applications, such as wireless communication and radar systems [4,5,6]. Fast beam steering, high reliability and multifunction capability are the main advantages that phased-array antennas can offer over traditional antenna systems [7]. Phased-array antennas and MIMO systems indeed offer practical advantages. Phased arrays improve communication bandwidth through beamforming, while enhancing radar accuracy with agile beam steering. These benefits vary across domains. In 5G, this means faster data rates; in radar, it means better tracking. The advantages extend to medical imaging and automotive radars. Each domain benefits uniquely from phased arrays based on its specific needs [8,9]. Each array element in the phased array is controlled in amplitude and phase with attenuators and phase shifters.

Massive MIMO is one of the major fifth-generation mobile communication technologies that can increase capacity by ten times or more, as well as provide high capacity with low latency [10,11,12]. Massive MIMO is an interdisciplinary technology that includes communication systems, signal processing and microwave technology [13,14]. In a recent effort to realize more spectacular gains and simplify the processing of required signals, massive MIMO systems were proposed in [15]. Each base station was equipped with more than 1 antenna, sometimes 100 or more [16]. However, many issues still need to be addressed for its practical implementation. The effect of correlation and mutual coupling on MIMO performance when additional antennas are arranged in the same physical space was investigated in [17] for a single-user case.

Due to some uncertainties and errors caused by mechanical manufacturing tolerance, namely variations in equipment temperature and electronic components, the system’s beampattern performance will degrade. Array uncertainties can be classified as geometrical or electrical. Geometrical uncertainties arise due to inaccurate spacing between antenna elements. Antenna elements are typically separated by half-wavelength distance or lower in order to minimize the array size [18,19]. On the other hand, electrical uncertainties occur because of imperfections in the electronics of the antenna array [20,21]. A small change in phase and amplitude can change the signal and degrade the performance of the system, which can increase the sidelobe, decrease the peak of the main lobe and/or change the direction of the main lobe. Thus, calibration is important to remove these uncertainties and to increase the accuracy of the system.

Another challenge regarding the electrical components is the nonlinearity of the transmit power amplifiers. For instance, most transmit array systems for radars operate at the saturated region of the amplifier, which imposes a distortion on the transmitted signals. Specifically, the power amplifier output is a clipped version of the input signal. To prevent this problem, the transmit waveforms should have a constant modulus constraint (CMC) or low peak-to-average ratio (PAR) [8,22]. Because of this, the calibration of the transmitter is more challenging than that of the receiver [23].

In the literature, the calibration of mutual coupling has been investigated for several decades. For microstrip patch antennas, various solutions are adopted to enhance the isolation of the antenna elements [24,25,26,27,28]. Some of the proposed techniques use purely theoretical assumptions to model the mutual coupling as an additional impedance. In [29], the authors used the antenna current Green function method to explain the mutual coupling interactions between the antennas and combine it with an electromagnetic machine learning algorithm to mitigate the effect of mutual coupling. In [30], the mutual coupling was analyzed by calculating the mutual impedance using the transmission coefficient measurement and knowledge of antenna distance. In [31,32], the author used the method of moments (MoM) for mutual coupling compensation on the receiver side. In [33], mutual coupling was studied and calibrated using measurement data recorded when the signal-to-noise ratio was high. This method can provide excellent results when the direction of the target is known. In [34], the author incorporated the reiterative super-resolution (RISR) algorithm into a calibration framework adapted to the estimation of mutual coupling. The algorithm is suitable for estimating the mutual coupling for a uniform, linear array and it depends on several sources and the signal-to-noise ratio (SNR). A significant challenge encountered in the management of multipath signals pertains to their substantial correlation with the line-of-sight (LOS) signal. Within the beamforming process, the existence of notable correlation between desired and undesired signals gives rise to signal cancellation phenomena, thereby exerting a deleterious impact on the performance of conventional beamforming techniques. This correlation-induced signal cancellation poses a significant impediment to the efficacy of beamforming methods commonly employed in practical applications [35,36]. Numerous applications spanning a wide range of fields, including but not limited to haptic feedback systems, telemedicine platforms and autonomous vehicles connected through networks, necessitate the need for swift and efficient calibration processes. These applications rely on precise and timely adjustments to ensure optimal performance, responsiveness and accuracy. Therefore, the ability to carry out calibration procedures quickly and effectively becomes a critical requirement for the successful operation and usability of these technologies [37,38]. In healthcare devices, mutual coupling can lead to inaccuracies in measurements, interference between sensors and reduced data reliability. These effects can compromise the diagnostic value and patient safety of these devices, highlighting the importance of addressing mutual coupling issues in their design and operation [39].

However, to the best of our knowledge, all the abovementioned methods use a directed matrix inversion to compute mutual coupling and, as a result, require high computational complexity. Moreover, these calibration methods use more than one source to estimate the mutual coupling. Furthermore, there is no method to calibrate the mutual coupling for large-scale antennas such as massive MISO systems. For massive MISO systems, our algorithm can achieve excellent performance, while the order of the computational complexity is very low and independent of the size of the antenna array.

### 1.1. Contributions

This paper’s main contributions can be summarized as follows:

- We estimate the mutual coupling of a transmit antenna array by developing a matrix-inversion-free algorithm. Most methods in the literature that estimate mutual coupling require an M×M matrix inversion, which may not be practical for a large antenna array.
- The algorithm requires a single antenna element at the receiver. Our method requires only one receiver with a known location to capture the the transmitted signals (training sequence) from the transmit array.
- The algorithm utilizes a constant modulus training sequence; thus, it can work at the saturated region of the high-power amplifier. High-power amplifiers, used in most active radars, work at the saturated region of the amplifiers.
- Our simulation results show the effectiveness of the developed algorithm in terms of fast estimation and excellent performance, even for large array systems such as massive MISO.
- The compensation complexity in our method is $M\log_{2}\left(M\right)+p^{3.5}$, whereas in the previous methods, it is stated to be $M^{3.5}$.

**Remark** **1.**

*1*: *In this paper, we focus on the mutual coupling on the transmitter side only. We assume that we have multiple antenna transmitters and only have a single antenna receiver.*
*2*: *The algorithm is also applicable to receiving antenna arrays. In this case, one or more single antenna transmitters are required. In this paper, we focus on the transmitter side only, since the transmit mutual coupling is much more challenging due to the CMC requirement described earlier [8].*
*3*: *This algorithm is useful for radar engineers and communication system designers.*

### 1.2. Notations

The notations used in this document are consistent with the following conventions. Vectors are denoted in bold, x (lower case), and matrices are denoted in bold, X (upper case). I(M) denotes the identity matrix of M×M. $x^{T}$ and $x^{H}$ represent transpose and Hermitian (the conjugate transpose) vectors, respectively.

## 2. Materials and Methods

**Figure 1:**
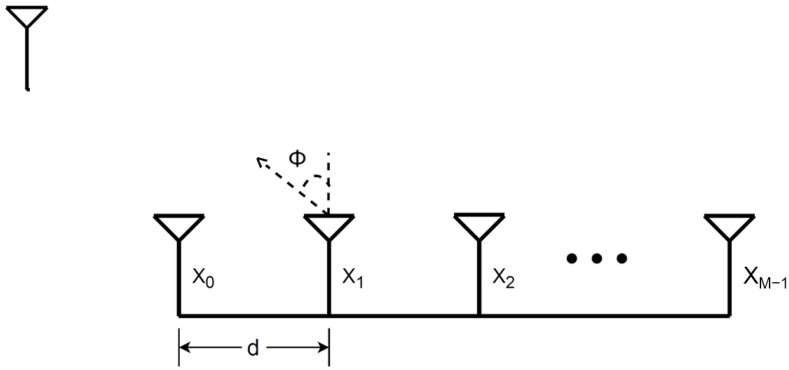
Uniform linear array antenna configuration.

In this section, we develop a method to calibrate the mutual coupling. This algorithm can calibrate the signal using only one external source. We consider a uniform linear array, as shown in Figure 1 and a narrowband *M*-element uniform linear array with a *d*-element spacing. The baseband signal is denoted by $x_{m}\left(t\right)$ and $f_{c}$ is the carrier frequency. The modulated signal on each channel is given by:

(1) $$x\left(t\right)=x_{m}\left(t\right)e^{j2\pi f_{c}t}$$

The sampled version of the $m^{th}$ transmitted baseband signal is denoted by $x_{m}\left(n\right)=x_{m}\left(t=nT_{s}\right)$, where *N* is the total number of time samples and $T_{s}=1/B$ is the sampling rate. Therefore, x is:

(2) $$x\left(n\right)={\left[x_{0}\left(n\right)\;x_{1}\left(n\right)\;\ldots \;x_{M-1}\left(n\right)\right]}^{T}$$

The mutual coupling matrix can be calculated using [40,41]:

(3) $$C=\left(Z_{A}+Z_{T}\right){\left(Z+Z_{T}I\right)}^{-1}=\left[\begin{array}{ccccc} C_{11} & C_{12} & \ldots & C_{1(M-2)} & C_{1(M-1)}\\ C_{21} & C_{22} & \ldots & C_{2(M-2)} & C_{2(M-1)}\\ \vdots & \; & \ddots & \; & \vdots \\ C_{(M-2)1} & C_{(M-2)2} & \ldots & C_{\left(M-2)(M-2\right)} & C_{\left(M-2)(M-1\right)}\\ C_{(M-1)1} & C_{(M-2)2} & \ldots & C_{\left(M-2)(M-1\right)} & C_{\left(M-1)(M-1\right)} \end{array}\right]$$

where $Z_{A}$ is the element’s impedance, $Z_{T}$ is the impedance of the receiver at each element and Z is the mutual impedance. Mutual coupling between antennas decreases as we move further away from the diagonal elements.

The mutual coupling matrix between the antennas is denoted by $C\in C^{M\times M}$, and we assume that the coupling extends to consecutive elements as expressed in Equation (3) [42,43,44].

(4) $$C=\left[\begin{array}{c} C_{ij} \end{array}\right]$$

where *i* is the element of the *i*th row and *j* is the element of the *j*th column. In the far-field, the received signal *r* can be expressed as:

(5) $$r\left(n\right)=x^{H}\left(n\right)\;C\;a\left(\phi \right)$$

where a is the steering vector shown in Equation (6):

(6) $$a\left(\phi \right)={\left[1\;e^{j\frac{2\pi }{\lambda }d\sin\left(\phi \right)}\;\ldots \;e^{j\frac{2\pi }{\lambda }d\sin\left(\phi \right)\left(M-1\right)}\right]}^{T}$$

where λ is the wavelength, *d* is the distance between the antennas and ϕ is the direction of the receiver such that $\left\{\phi \;|\;-90^{\circ }\leq \phi \leq 90^{\circ }\right\}$. Let X be the transmitted signal at *M* different times, so X can be represented as:

(7) $$X=\left[\begin{array}{c} X_{kl} \end{array}\right]$$

where *k* is the element of the *k*th row and *l* is the element of the *l*th column. Therefore, the received signals will be a vector of size M×1, as shown below:

(8) r=XCa(ϕ)

(9) $$r\left(n\right)={\left[r\left(0\right)\;r\left(1\right)\;\ldots \;r\left(M-1\right)\right]}^{T}$$

We set X to be the discrete Fourier transform (DFT) matrix, as shown in Equation (10) and we will use N=M to reduce and accelerate the computations. The total number of complex multiplications and additions in the calculation of all *M* DFT samples is equal to $M\log_{2}\left(M\right)$ [45]. Furthermore, it is easy to find the inverse of the DFT [46,47].

(10) $$X=\left[\begin{array}{ccccc} 1 & 1 & 1 & \ldots & 1\\ 1 & e^{\frac{2\pi i}{M}} & e^{\frac{2\pi i(2)}{M}} & \ldots & e^{\frac{2\pi i(M-1)}{M}}\\ 1 & e^{\frac{2\pi i(2)}{M}} & e^{\frac{2\pi i(4)}{M}} & \ldots & e^{\frac{2\pi i(2)(M-1)}{M}}\\ \vdots & \vdots & \ddots & \ddots & \vdots \\ 1 & e^{\frac{2\pi i(M-1)}{M}} & e^{\frac{2\pi i(2)(M-1)}{M}} & \ldots & e^{\frac{2\pi i(M-1)(M-1)}{M}} \end{array}\right]$$

In this case, the received signals can be represented as:

(11) r=FFT(Ca(ϕ))

FFT stands for fast Fourier transform, which is used to compute the multiplication of the DFT matrix X using the vector Ca(ϕ). Similarly, IFFT stands for inverse fast Fourier transform, which is used to compute the multiplication of the inverse DFT matrix of X using the vector r. If we use the direct multiplication in Equation (8), the computational complexity will be proportional to $M^{2}$. However, using FFT to compute the same quantity requires only $M\log_{2}\left(M\right)$ utilizing the structure of the DFT matrix in Equation (10) [46,47]. Thus, we can rewrite the previous equation as:

(12) $$C^{-1}IFFT\left(r\right)=C^{-1}b=a\left(\phi \right)$$

where **b** = IFFT(r). The matrix C has consecutive elements, as described before in Equation (3). Therefore, the inverse of the mutual coupling matrix will also have a specific configuration and the matrix configuration will be a symmetric Toeplitz matrix with *M* unknown elements.

(13) $$\begin{array}{c} C^{-1}=\frac{1}{\left(Z_{A}+Z_{T}\right)}\left(Z+Z_{T}I\right) \end{array}$$

**Figure 2:**
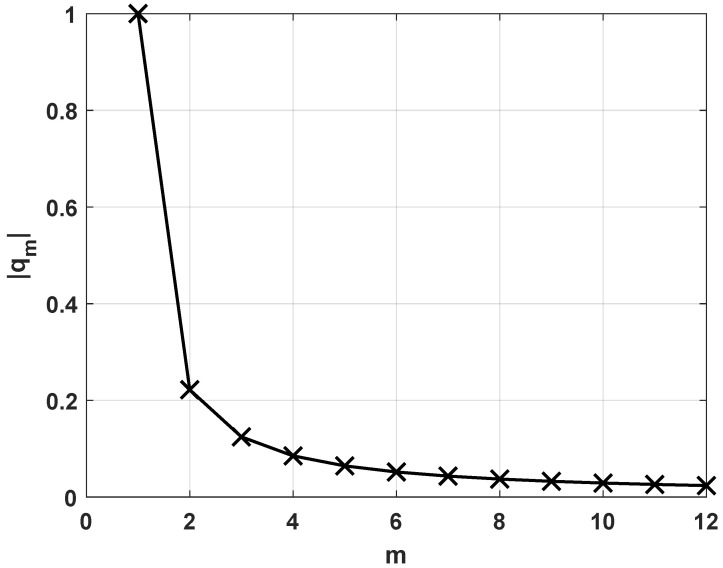
Magnitude of the normalized inverse of mutual coupling between array elements.

For linear geometries, Figure 2 illustrates the magnitude of the normalized mutual coupling components for an array of M=12 dipoles with a spacing of l=λ/2 and terminating impedance $Z_{T}=Z_{A}$. It shows that the inverse of mutual coupling between nearby elements in a linear array is almost the same. As we move further away from the diagonal, the amplitude of the inverse of the mutual coupling decreases significantly.

From the matrix in Equation (12), we observe that the inverse of the mutual coupling matrix $C^{-1}$ has *M* unknown elements. Thus, our system can be represented as:

(14) $$C^{-1}b=a\left(\phi \right)=\underset{C^{-1}}{\underset{︸}{\left[\begin{array}{cccc} q_{1} & q_{2} & \ldots & q_{M}\\ q_{2} & q_{1} & \ldots & q_{M-1}\\ q_{3} & q_{2} & \ldots & q_{M-2}\\ \vdots & \vdots & \ddots & \vdots \\ q_{M} & q_{M-1} & \ldots & q_{1} \end{array}\right]}}\underset{b}{\underset{︸}{\left[\begin{array}{c} b_{1}\\ b_{2}\\ b_{3}\\ \vdots \\ b_{M} \end{array}\right]}}=\underset{a(\phi )}{\underset{︸}{\left[\begin{array}{c} a_{1}\left(\phi \right)\\ a_{2}\left(\phi \right)\\ a_{3}\left(\phi \right)\\ \vdots \\ a_{M}\left(\phi \right) \end{array}\right]}}$$

Equation (14) represents a system of equations with *M* unknowns and equations, which can be written as:

(15) $$\begin{array}{cc} b_{1}q_{1}+b_{2}q_{2}+b_{3}q_{3}+\ldots +b_{M}q_{M} & =a_{1}\left(\phi \right)\\ b_{1}q_{2}+b_{2}q_{1}+b_{3}q_{2}+\ldots +b_{M}q_{\left(M-1\right)} & =a_{2}\left(\phi \right)\\ b_{1}q_{3}+b_{2}q_{2}+b_{3}q_{1}+\ldots +b_{M}q_{\left(M-2\right)} & =a_{3}\left(\phi \right)\\ \vdots \\ b_{1}q_{M}+b_{2}q_{\left(M-1\right)}+b_{3}q_{\left(M-2\right)}+\ldots +b_{M}q_{1} & =a_{M}\left(\phi \right) \end{array}$$

Our objective is to identify and determine the values of the unknown elements in the inverse mutual coupling matrix $C^{-1}$ (i.e., the values of $q_{1},\;q_{2},\;\ldots ,$ and $\;q_{M}$). To achieve this, we assign the unknown elements to the corresponding elements in a vector denoted by q:

(16) $$q={\left[q_{1}\;q_{2}\;q_{3}\;\ldots \;q_{\left(M-2\right)}\;q_{\left(M-1\right)}\;q_{M}\right]}^{T}$$

Notice that in the second line in (15), the first and third terms have $q_{2}$. Similarly, in the third line, the first and fifth terms have $q_{3}$ and the second and fourth terms have $q_{2}$. Then, Equation (15) can be solved to obtain the values of the unknown elements.

(17) $$\underset{B}{\underset{︸}{\left[\begin{array}{cccc} b_{1} & b_{2} & \ldots & b_{M}\\ b_{2} & b_{1}+b_{3} & \ldots & 0\\ b_{3} & b_{2}+b_{4} & \ldots & 0\\ \vdots & \vdots & \ddots & \vdots \\ b_{\left(M-2\right)} & b_{\left(M-3\right)}+b_{\left(M-1\right)} & \ldots & 0\\ b_{\left(M-1\right)} & b_{2}+b_{M} & \ldots & 0\\ b_{M} & b_{\left(M-1\right)} & \ldots & b_{1} \end{array}\right]}}\underset{q}{\underset{︸}{\left[\begin{array}{c} q_{1}\\ q_{2}\\ q_{3}\\ \vdots \\ q_{\left(M-2\right)}\\ q_{\left(M-1\right)}\\ q_{M} \end{array}\right]}}=\underset{a(\phi )}{\underset{︸}{\left[\begin{array}{c} a_{1}\left(\phi \right)\\ a_{2}\left(\phi \right)\\ a_{3}\left(\phi \right)\\ \vdots \\ a_{M}\left(\phi \right) \end{array}\right]}}$$

(18) Bq=a(ϕ)

To reduce computations, we aim to decrease the number of unknown elements *q* to *p*, where *p* is less than *M*. By reducing *p*, we optimize the system and can estimate the mutual coupling with fewer multiplications, additions and inversions. In systems with a large number of antennas, it is desirable to calibrate the system using the smallest possible value of *p*.

The modified inverse mutual coupling matrix ${\hat{C}}^{-1}$, when p<M, can be expressed as:

(19) $${\hat{C}}^{-1}=\left[\begin{array}{cccccc} q_{1} & \ldots & q_{p} & 0 & \ldots & 0\\ \vdots & \ddots & \vdots & q_{p} & \ddots & \vdots \\ q_{p} & \vdots & q_{1} & \vdots & \ddots & 0\\ 0 & \ddots & \vdots & q_{1} & \ldots & q_{p}\\ \vdots & \ddots & q_{p} & \vdots & \ddots & \vdots \\ 0 & \ldots & 0 & q_{p} & \ldots & q_{1} \end{array}\right]$$

As the number of unknown elements in $C^{-1}$ is reduced, Equations (14) and (15) are modified accordingly.

(20) $${\hat{C}}^{-1}b=a\left(\phi \right)=\left[\begin{array}{cccccc} q_{1} & \ldots & q_{p} & 0 & \ldots & 0\\ \vdots & \ddots & \vdots & q_{p} & \ddots & \vdots \\ q_{p} & \vdots & q_{1} & \vdots & \ddots & 0\\ 0 & \ddots & \vdots & q_{1} & \ldots & q_{p}\\ \vdots & \ddots & q_{p} & \vdots & \ldots & \vdots \\ 0 & \ldots & 0 & q_{p} & \ldots & q_{1} \end{array}\right]\left[\begin{array}{c} b_{1}\\ b_{2}\\ b_{3}\\ \vdots \\ b_{M} \end{array}\right]=\left[\begin{array}{c} a_{1}\left(\phi \right)\\ a_{2}\left(\phi \right)\\ a_{3}\left(\phi \right)\\ \vdots \\ a_{M}\left(\phi \right) \end{array}\right]$$

(21) $$\begin{array}{cc} b_{1}q_{1}+b_{2}q_{2}+b_{3}q_{3}\;\cdots +b_{p}q_{p} & =a_{1}\left(\phi \right)\\ b_{1}q_{2}+b_{2}q_{1}+b_{3}q_{2}\;\cdots +b_{\left(p+1\right)}q_{p} & =a_{2}\left(\phi \right)\\ b_{1}q_{3}+b_{2}q_{2}+b_{3}q_{1}\;\cdots +b_{\left(p+2\right)}q_{p} & =a_{2}\left(\phi \right)\\ \vdots \\ b_{\left(M-p+1\right)}q_{p}+b_{\left(M-p+2\right)}q_{\left(p-1\right)}\;\cdots +b_{M}q_{1} & =a_{M}\left(\phi \right) \end{array}$$

Again, the process is similar to that in (17) after modifying $C^{-1}$. This reduces the size of B to M×p instead of M×M, denoted by $\hat{B}$ and the unknown elements are denoted by $\hat{q}$ with a size of p×1, as shown in Equations (22) and (23).

(22) $$\hat{B}=\left[\begin{array}{cccc} b_{1} & b_{2} & \ldots & b_{p}\\ b_{2} & b_{1}+b_{3} & \ldots & b_{\left(p+1\right)}\\ b_{3} & b_{2}+b_{4} & \ldots & b_{\left(p+2\right)}\\ \vdots & \vdots & \ddots & \vdots \\ b_{\left(M-2\right)} & b_{\left(M-3\right)}+b_{\left(M-1\right)} & \ldots & b_{\left(M-p+3\right)}\\ b_{\left(M-1\right)} & b_{2}+b_{M} & \ldots & b_{\left(M-p+2\right)}\\ b_{M} & b_{\left(M-1\right)} & \ldots & b_{\left(M-p+1\right)} \end{array}\right]$$

(23) $$\hat{q}={\left[\begin{array}{ccccccc} q_{1} & q_{2} & q_{3} & \ldots & q_{\left(p-2\right)} & q_{\left(p-1\right)} & q_{p} \end{array}\right]}^{T}$$

Consequently, Equation (24) requires significantly fewer computations compared to Equation (18).

(24) $$\hat{B}\hat{q}=\;a\left(\phi \right)$$

When the number of equations is greater than the number of unknowns, it is called an overdetermined system. To solve the system and estimate the inverse mutual coupling using the least-squares method [48], we use

(25) $$\hat{q}={\left({\hat{B}}^{H}\hat{B}\right)}^{-1}{\hat{B}}^{H}a\left(\phi \right)$$

Once the estimation is calculated, it can be used to eliminate mutual coupling, where the transmit signal is designed as $x^{H}{\hat{C}}^{-1}$:

(26) $$r=x^{H}{\hat{C}}^{-1}Ca\left(\phi \right)$$

From a mathematical perspective, the number of multiplications required to obtain the product of a matrix grows much faster than the number of additions. Thus, reducing the number of elements from *M* to *p* not only reduces the size of the matrix, but also leads to computational advantages.

The algorithm is presented in Algorithm 1, where it outlines the steps for estimating the inverse of mutual coupling ${\hat{C}}^{-1}$ using the following given inputs: **r** (the received signal), a(ϕ) (the steering vector), $\hat{q}$ (a vector containing the unknown elements of ${\hat{C}}^{-1}$) and p<M. The computational complexity of Algorithm 1 is $O(M\log_{2}\left(M\right)+p^{3.5})$, where p<<M.
**Algorithm 1:** Mutual coupling algorithm**Input:** X, r, a(ϕ), $\hat{q}$ and p<MWhere X is Discrete Fourier Transform (DFT) matrix,r is the received signala(ϕ) is the steering vector$\hat{q}$ is a vector containing the unknown elements of ${\hat{C}}^{-1}$**Output:** ${\hat{C}}^{-1}$ (Inverse of mutual coupling)(1) Send X as DFT matrix(2) Find **b** = IFFT(**r**), where r=XCa(ϕ)(3) Construct ${\hat{C}}^{-1}$ using Equation (19), based on *p* unknown elements(4) Using Equations (20) and (21), construct $\hat{B}$ using Equation (22)(5) Find $\hat{q}={\left({\hat{B}}^{H}\hat{B}\right)}^{-1}{\hat{B}}^{H}a\left(\phi \right)$(6) Construct ${\hat{C}}^{-1}$ using Equation (19)

The computational complexity of Algorithm 1 is given by $O(M\log_{2}\left(M\right)+p^{3.5})$, where p<<M.

## 3. Results

In this section, we propose an algorithm to calculate the inverse of the mutual coupling for different numbers of antennas. We used MATLAB software to verify the efficiency of the proposed approach. The simulations encompass two scenarios. The first scenario involves variations in signal-to-noise ratios (SNR) ranging from low to high, while the second scenario focuses on the signal-to-interference-noise ratio (SINR). To reduce the effect of noise, we sent the signals four times and took the average. We considered linear arrays of half-wavelength dipoles operating at 3 GHz, where the analytic formulas for mutual coupling are well known. The desired beampattern was directed at −5° degrees with multiple nulls. We zoomed in on the range from 15 to 25 degrees to provide a clearer depiction of the null in this region. Throughout the simulation, we used a uniform linear array with a distance of λ/2 between antennas and there was one external source at −5°. All the parameter values used in the simulations are listed in Table 1.

We chose a small value for *p* to reduce the complexity of the algorithm. Increasing the value of *p* would result in an increase in the algorithm’s complexity.

### 3.1. Low Signal-to-Noise Ratio (SNR)

**Figure 3:**
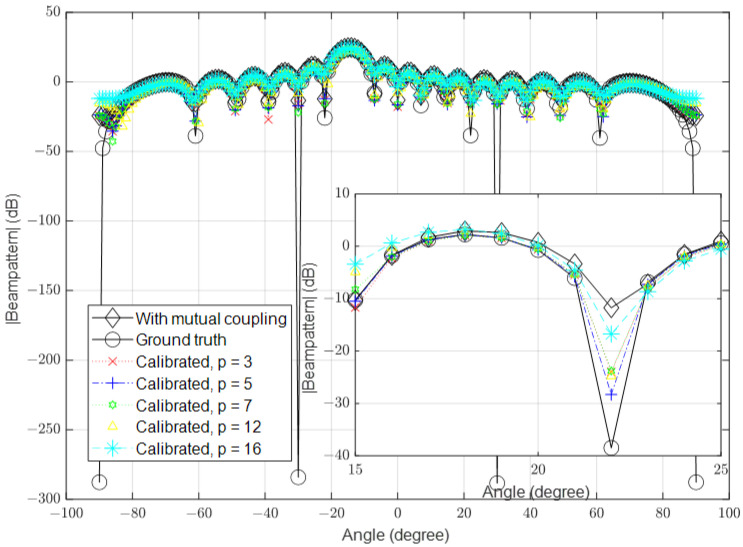
Calibrated signal using different values of *p* when the SNR is low and M=16, compared to the ground truth and uncalibrated signals.

**Figure 4:**
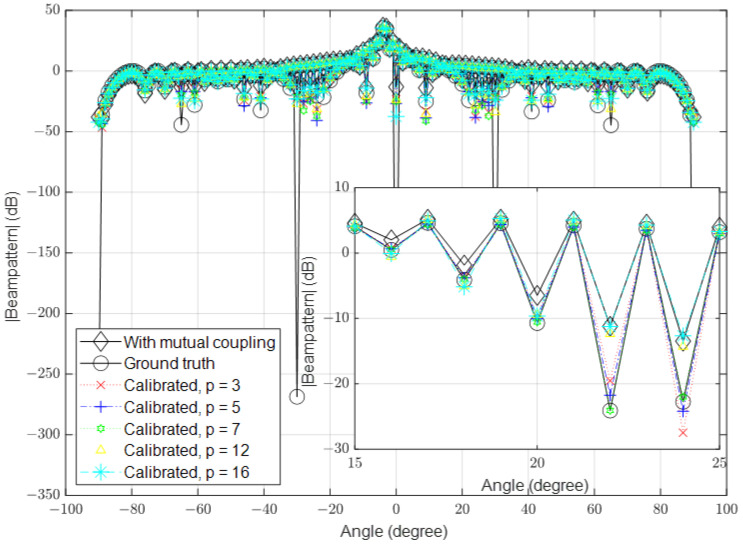
Calibrated signal using different values of *p* when the SNR is low and M=64, compared to the ground truth and uncalibrated signals.

**Figure 5:**
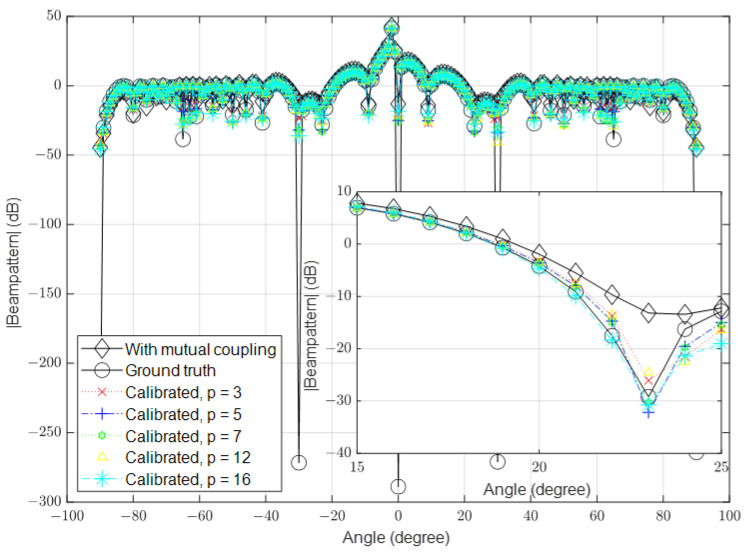
Calibrated signal using different values of *p* when the SNR is low and M=128, compared to the ground truth and uncalibrated signals.

In this part, we simulate the algorithm under low-SNR conditions, where the noise is high in some channels. In the first simulation, we set the number of antennas to 16 and the SNR to 10 dB. The mutual coupling was calculated using Equation (3). In the second simulation, we increased the number of antennas to 64 and in the third simulation, we further increased it to 128. The spatial spectrum for the received signal in the presence of mutual coupling, the ground-truth received signal (when the mutual coupling was absent and the noise was ignored) and the calibrated signals with different values of *p* are shown in Figure 3, Figure 4 and Figure 5, respectively.

### 3.2. High Signal-to-Noise Ratio (SNR)

**Figure 6:**
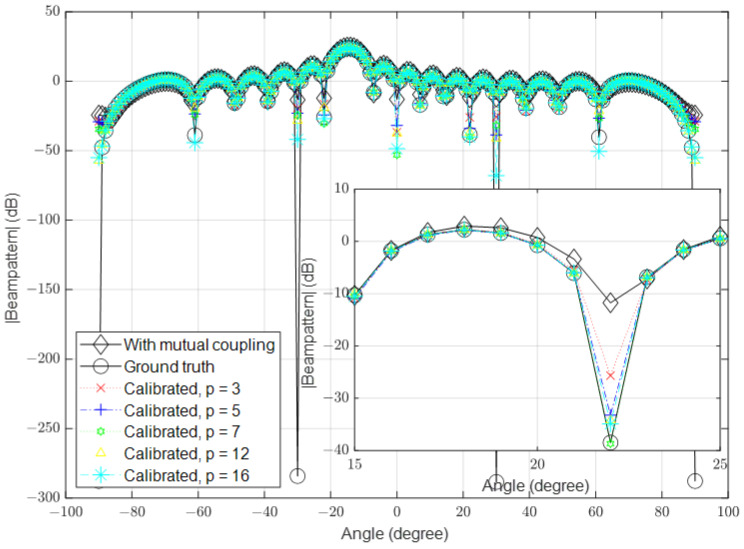
Calibrated signal using different values of *p* when the SNR is high and M=16, compared to the ground truth and uncalibrated signals.

**Figure 7:**
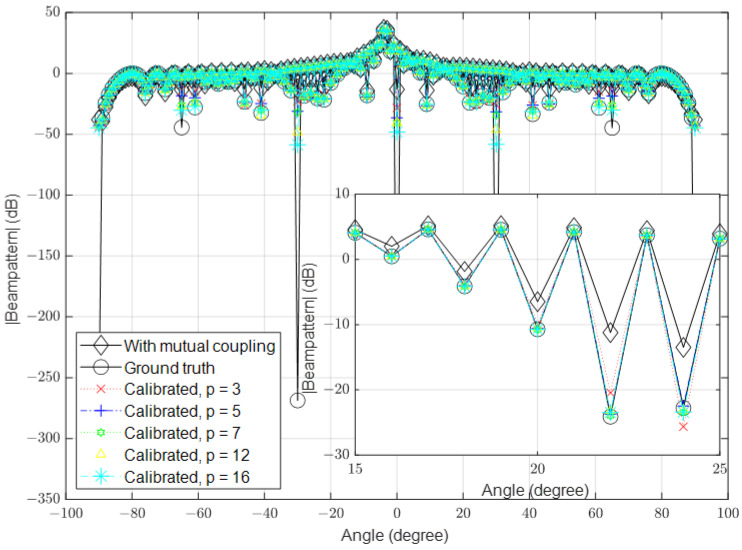
Calibrated signal using different values of *p* when the SNR is high and M=64, compared to the ground truth and uncalibrated signals.

**Figure 8:**
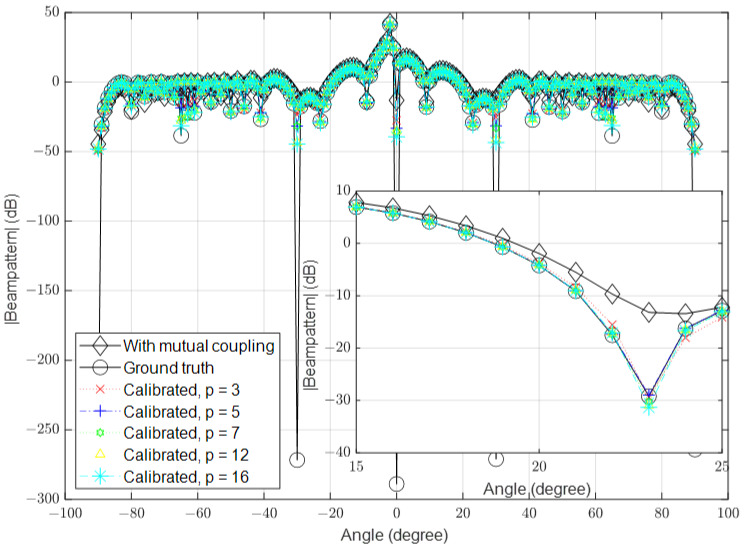
Calibrated signal using different values of *p* when the SNR is high and M=128, compared to the ground truth and uncalibrated signals.

In this part, we simulate the algorithm under high-SNR conditions. Similar to the previous part, we set the number of antennas to 16, 64 and 128 and the SNR to 40 dB in the different simulations. The results for these scenarios are shown in Figure 6, Figure 7 and Figure 8, respectively.

### 3.3. Multipath Environment Scenario

**Figure 9:**
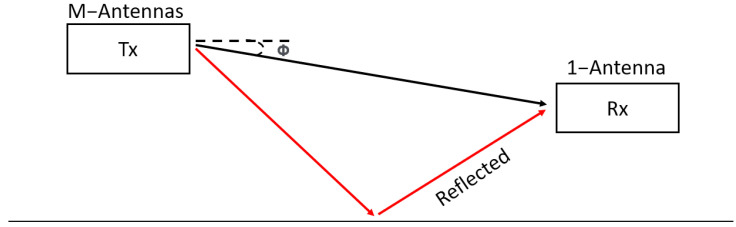
Multipath fading scenario from flat surface.

In this section, a multipath scenario was simulated. In this scenario, a line-of-sight (LoS) path and one reflected path from a flat surface were simulated as shown in Figure 9.

**Figure 10:**
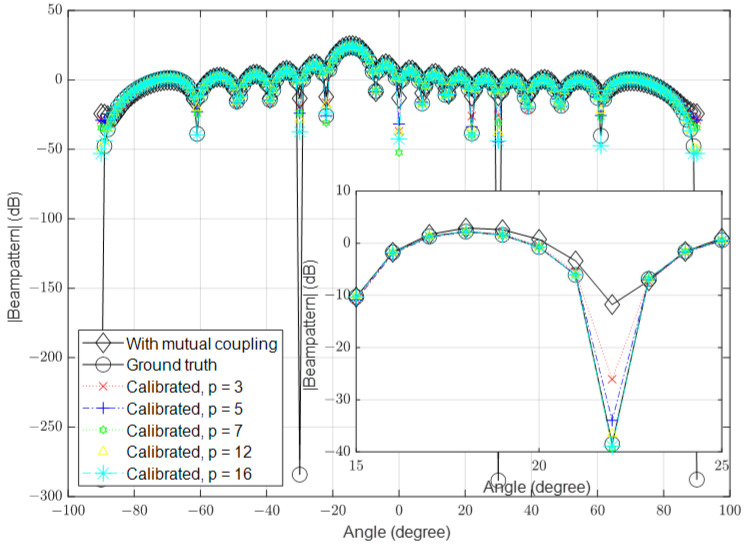
Calibrated signal using different values of *p* when the SNR is high and M=16, compared to the ground truth and uncalibrated signals when SINR is present.

**Figure 11:**
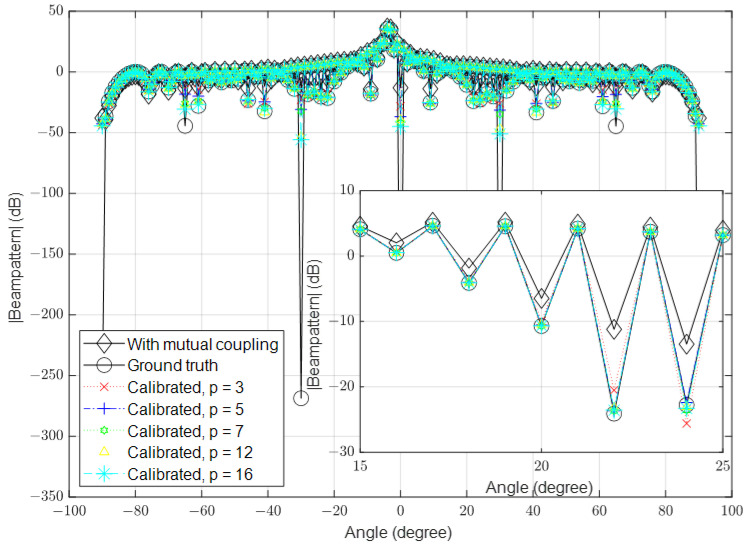
Calibrated signal using different values of *p* when the SNR is high and M=64, compared to the ground truth and uncalibrated signals when SINR is present.

**Figure 12:**
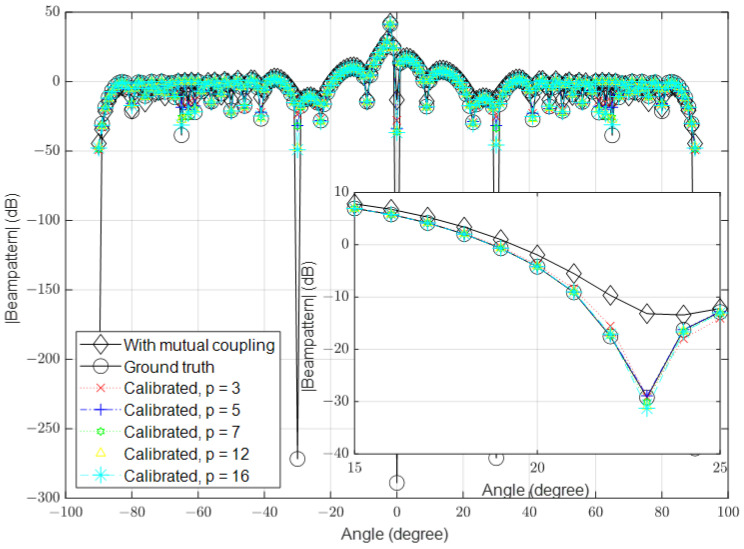
Calibrated signal using different values of *p* when the SNR is high and M=128, compared to the ground truth and uncalibrated signals when SINR is present.

Similar to the previous part, we set the number of antennas to 16, 64 and 128, the SNR is set to 40 dB and the reflected signal is equal to 4% of direct signal in the different simulations. The calibrated signals with different values of p are shown in Figure 10, Figure 11 and Figure 12, respectively.

The results demonstrate that the proposed algorithm achieves excellent accuracy in mutual coupling estimation. It is evident that the system can calibrate the effect of mutual coupling even when p=3, similar to the case when p=16. When the SNR is low, there is a small difference between the ground truth and the calibrated signals. The algorithm demonstrates the ability to calibrate mutual coupling in massive MISO systems when p=3, even in the presence of SINR.

The results indicate that the proposed algorithm provides efficient calibration even with increasing numbers of antennas. The time required to calculate the unknown vector $\hat{q}$ increases with the number of antennas, but it remains manageable. The compensation complexity in our proposed method is given by $M\log_{2}\left(M\right)+p^{3.5}$, whereas in reference [30,31,33], it is stated to be $M^{3.5}$. It is observed that as the value of M increases, the compensation complexity in our method also increases, but not significantly. In contrast, the complexity in the referenced method increases rapidly with an increasing M. See Table 2.

In Table 3, we compare the computations required in the proposed method when p=3 with computations required in previous method. It is evident that as the value of *M* increases, the number of computations in the previous method rises rapidly. In contrast, our method maintains a significantly lower number of computations when compared to the previous method.

The algorithm works very well for any uniform linear and circular array of any size, as there are no restrictions on the steering vector “a(ϕ)”. However, since the λ/2 uniform linear array is the most common for its versatility, we used it as an example. The algorithm works as long as the mutual coupling matrix is Toeplitz. For the memory requirement, the number of variables needed to be stored is at most 2(M+p), where *p* is the number of distinct variables in the M×M mutual coupling matrix (usually p<<M) and *M* is the variable of the steering vector. Since we are dealing with complex numbers, each variable is multiplied by two to account for the real and imaginary parts [49].

## 4. Conclusions

Mutual coupling was calculated for a half-dipole ULA based on basic electromagnetic concepts. As the mutual coupling often is unknown and needs to be estimated, a simpler method was proposed based on the FFT matrix. However, our methodology can estimate the mutual coupling for massive MISO systems. It is acceptable even when *p* is lower than M. Furthermore, our methodology can also be advantageous in terms of reduced computing time and required memory because it uses an FFT matrix. The advantages of the presented algorithm are of significance. Its efficacy with a solitary, known external source simplifies deployment. Furthermore, its computational efficiency, which maintains feasibility as antenna numbers increase, bears particular relevance within the domain of 5G. This is crucial given the imperative to optimize network performance and beamforming strategies in this context. Simulation results support our analysis and demonstrate the algorithm’s calibration performance on the beampattern, even with a small *p* value. So far, simulation results have shown an excellent performance in all the tested scenarios. In future work, considering mutual coupling at the receiver will be essential for assessing and enhancing the algorithm’s performance and validity.

## Figures and Tables

**Table 1:** Algorithm parameters.

Parameter	Value
*f*	3 GHz
λ	10 cm
*d*	$\frac{\lambda }{2}$
Number of antennas (M)	16, 64, 128
*p*	3, 5, 7, 12, 16
Direction of source	−5°

**Table 2:** Comparison method.

Method	Compensation Complexity	Number of Receivers or Transmitters	Receiver or Transmitter Side
Method in [30]	$M^{3.5}$	1	Transmitter or receiver
Method in [31]	$M^{3.5}$	1	Receiver
Method in [33]	$M^{3.5}$	1 (records a lot of data)	Receiver
Proposed method	$M\;\log_{2}\left(M\right)$ + $p^{3.5}$	1	Transmitter (can be extended to receiver)

**Table 3:** The number of computations (number of multiplications and additions) to calculate the mutual coupling matrix when p=3.

Number of Antennas (M)	Computations Required in [30,31,33]	Computations Required for the the Proposed Method
4	128	55
16	16,384	110
64	2,097,152	431
128	2.37 × 10${}^{7}$	943

## Data Availability

No new data were created or analyzed in this study. Data sharing is not applicable to this article.

## Abbreviations

The following abbreviations are used in this manuscript:

MISO	Multi-input–single-output
MIMO	Multi-input–multi-output
CMC	constant modulus constraint
PAR	Peak-to-average ratio
MOM	Method of moments
RISR	Reiterative super-resolution
DFT	Discrete Fourier transform
FFT	Fast Fourier transform
IFFT	Inverse Fast Fourier transform
SNR	Signal-to-noise ratio
LOS	Line of sight
SINR	Signal-to-interference-noise ratio

## References

[1] Gross F. (2005). Smart Antennas for Wireless Communications with MATLAB.

[2] Widrow B., Stearns S.D., Burgess J.C. (1986). Adaptive signal processing edited by bernard widrow and samuel d. stearns. J. Acoust. Soc. Am..

[3] Biglieri E., Calderbank R., Constantinides A., Goldsmith A., Paulraj A., Poor H.V. (2007). MIMO Wireless Communications.

[4] Willerton M., Manikas A. Virtual linear array modelling of a planar array. Proceedings of the 2nd IMA Conference on Mathematics in Defence.

[5] Manikas A. (2004). Differential Geometry in Array Processing.

[6] Xin Y., Yang L., Wang D., Zhang R., You X. (2017). Bidirectional dynamic networks with massive MIMO: Performance analysis. IET Commun..

[7] Medina-Sanchez R.H. (2014). Beam Steering Control System for Low-Cost Phased Array Weather Radars: Design and Calibration Techniques. Doctoral Thesis.

[8] He H., Stoica P., Li J. (2010). Wideband MIMO systems: Signal design for transmit beampattern synthesis. IEEE Trans. Signal Process..

[9] Sharma O.K., Shankar T., Krishnatre P. Reducing interference of Gaussian MIMO Z channel and Gaussian MIMO X Channel. Proceedings of the IEEE International Conference on Engineering and Technology (ICETECH).

[10] Larsson E.G., Edfors O., Tufvesson F., Marzetta T.L. (2014). Massive MIMO for next generation wireless systems. IEEE Commun. Mag..

[11] Narukawa N., Fukushima T., Honda K., Ogawa K. 64 × 64 MIMO antenna arranged in a daisy chain array structure at 50 Gbps capacity. Proceedings of the 2019 URSI International Symposium on Electromagnetic Theory (EMTS).

[12] Yamaguchi S., Nakamizo H., Shinjo S., Tsutsumi K., Fukasawa T., Miyashita H. Development of active phased-array antenna for high SHF wideband massive MIMO in 5G. Proceedings of the 2017 IEEE International Symposium on Antennas and Propagation & USNC/URSI National Radio Science Meeting.

[13] Yuan H., Wang C., Li Y., Liu N., Cui G. The design of array antennas used for Massive MIMO system in the fifth generation mobile communication. Proceedings of the 2016 11th International Symposium on Antennas, Propagation and EM Theory (ISAPE).

[14] Won S.H., Chae S.C., Cho S.Y., Kim I., Bang S.C. Massive MIMO test-bed design for next-generation long term evolution (LTE) mobile systems in the frequency division duplex (FDD) mode. Proceedings of the 2014 International Conference on Information and Communication Technology Convergence (ICTC).

[15] Marzetta T.L. (2010). Noncooperative cellular wireless with unlimited numbers of base station antennas. IEEE Trans. Wirel. Commun..

[16] Lu L., Li G.Y., Swindlehurst A.L., Ashikhmin A., Zhang R. (2014). An overview of massive MIMO: Benefits and challenges. IEEE J. Sel. Top. Signal Process..

[17] Shen S., McKay M.R., Murch R.D. MIMO systems with mutual coupling: How many antennas to pack into fixed-length arrays?. Proceedings of the 2010 International Symposium On Information Theory & Its Applications.

[18] Thet N.W.M., Khan S., Arvas E., Özdemir M.K. (2020). Impact of mutual coupling on power-domain non-orthogonal multiple access (NOMA). IEEE Access.

[19] Ludwig A. (1976). Mutual coupling, gain and directivity of an array of two identical antennas. IEEE Trans. Antennas Propag..

[20] Akindoyin A. Modelling and estimation of carrier frequency and phase uncertainties in large aperture arrays. Proceedings of the IEEE International Conference on Communications.

[21] Willerton M. (2013). Array Auto-Calibration. Doctoral Thesis.

[22] Patton L., Rigling B. Modulus constraints in adaptive radar waveform design. Proceedings of the 2008 IEEE Radar Conference.

[23] Liao W.-C., Maaskant R., Emanuelsson T., Vilenskiy A., Ivashina M. Antenna mutual coupling effects in highly integrated transmitter arrays. Proceedings of the 2020 14th European Conference on Antennas and Propagation (EuCAP).

[24] Vishvaksenan K.S., Mithra K., Kalaiarasan R., Raj K.S. (2017). Mutual coupling reduction in microstrip patch antenna arrays using parallel coupled-line resonators. IEEE Antennas Wirel. Propag. Lett..

[25] Qamar Z., Naeem U., Khan S.A., Chongcheawchamnan M., Shafique M.F. (2016). Mutual coupling reduction for high-performance densely packed patch antenna arrays on finite substrate. IEEE Trans. Antennas Propag..

[26] Cheng Y.F., Ding X., Shao W., Wang B.Z. (2016). Reduction of mutual coupling between patch antennas using a polarization-conversion isolator. IEEE Antennas Wirel. Propag. Lett..

[27] Si L., Jiang H., Lv X., Ding J. (2019). Broadband extremely close-spaced 5G MIMO antenna with mutual coupling reduction using metamaterial-inspired superstrate. Opt. Express.

[28] Khan A., Bashir S., Ghafoor S., Rmili H., Mirza J., Ahmad A. (2023). Isolation Enhancement in a Compact Four-Element MIMO Antenna for Ultra-Wideband Applications. Cmc-Comput. Mater. Contin..

[29] Alzahed A.M., Mikki S.M., Antar Y.M. (2019). Nonlinear mutual coupling compensation operator design using a novel electromagnetic machine learning paradigm. IEEE Antennas Wirel. Propag. Lett..

[30] Vo T.T., Ouvry L., Sibille A., Bories S. Mutual Coupling Modeling and Calibration in Antenna Arrays for AOA Estimation. Proceedings of the 2018 2nd URSI Atlantic Radio Science Meeting (AT-RASC).

[31] Khan S., Sajjad H., Ozdemir M.K., Arvas E. Mutual coupling compensation in receiving antenna arrays. Proceedings of the 2020 International Applied Computational Electromagnetics Society Symposium (ACES).

[32] Khan S. (2021). Mutual Coupling Compensation in Arrays and its Implementation on Software Defined Radios. Ph.D. Thesis.

[33] Huang J.H., Garry J.L., Smith G.E., Tan D.K. In-field calibration of passive array receiver using detected target. Proceedings of the 2018 IEEE Radar Conference (RadarConf18).

[34] Cordill B.D., Seguin S.A., Blunt S.D. Mutual coupling calibration using the Reiterative Superresolution (RISR) algorithm. Proceedings of the 2014 IEEE Radar Conference.

[35] Daneshmand S., Broumandan A., Sokhandan N., Lachapelle G. (2013). GNSS multipath mitigation with a moving antenna array. IEEE Trans. Aerosp. Electron. Syst..

[36] Widrow B., Duvall K., Gooch R., Newman W. (1982). Signal cancellation phenomena in adaptive antennas: Causes and cures. IEEE Trans. Antennas Propag..

[37] Khan L.U., Yaqoob I., Imran M., Han Z., Hong C.S. (2020). 6G wireless systems: A vision, architectural elements and future directions. IEEE Access.

[38] Eltayeb M.E., Al-Naffouri T.Y., Heath R.W. Compressive sensing for blockage detection in vehicular millimeter wave antenna arrays. Proceedings of the IEEE Global Communications Conference (GLOBECOM).

[39] Kim J.H., Cho S.I., Kim H.J., Choi J.W., Jang J.E., Choi J.P. Mutual coupling in RF powered wireless communication networks for health monitoring. Proceedings of the IEEE International Workshop on Electromagnetics: Applications and Student Innovation Competition (iWEM).

[40] Balanis C.A. (1992). Antenna theory: A review. Proc. IEEE.

[41] Svantesson T. Modeling and estimation of mutual coupling in a uniform linear array of dipoles. Proceedings of the 1999 IEEE International Conference on Acoustics, Speech and Signal Processing. Proceedings. ICASSP99 (Cat. No. 99CH36258).

[42] Malanowski M., Kulpa K. Digital beamforming for passive coherent location radar. Proceedings of the 2008 IEEE Radar Conference.

[43] Steyskal H., Herd J.S. (1990). Mutual coupling compensation in small array antennas. IEEE Trans. Antennas Propag..

[44] Durrani S., Bialkowski M.E. (2004). Effect of mutual coupling on the interference rejection capabilities of linear and circular arrays in CDMA systems. IEEE Trans. Antennas Propag..

[45] Cooley J.W., Lewis P.A., Welch P.D. (1967). Historical notes on the fast Fourier transform. Proc. IEEE.

[46] Mitra S.K., Kuo Y. (2006). Digital Signal Processing: A Computer-Based Approach.

[47] Rao K.R., Yip P.C. (2018). The Transform and Data Compression Handbook.

[48] Williams G. (1990). Overdetermined systems of linear equations. Am. Math. Mon..

[49] Deng J., Wang Q., Xie J. Fourth-order cumulant based direction finding algorithm for non-circular signals using uniform circular array with mutual coupling. Proceedings of the IEEE International Conference on Signal Processing, Communications and Computing (ICSPCC).

